# What does spatial alternation tell us about retrosplenial cortex function?

**DOI:** 10.3389/fnbeh.2015.00126

**Published:** 2015-05-18

**Authors:** Andrew J. D. Nelson, Anna L. Powell, Joshua D. Holmes, Seralynne D. Vann, John P. Aggleton

**Affiliations:** School of Psychology, Cardiff UniversityCardiff, UK

**Keywords:** cingulate cortex, spatial memory, hippocampus, dementia, anterior thalamus, navigation

## Abstract

The retrosplenial cortex supports navigation, but there are good reasons to suppose that the retrosplenial cortex has a very different role in spatial memory from that of the hippocampus and anterior thalamic nuclei. For example, retrosplenial lesions appear to have little or no effect on standard tests of spatial alternation. To examine these differences, the current study sought to determine whether the retrosplenial cortex is important for just one spatial cue type (e.g., allocentric, directional or intra-maze cues) or whether the retrosplenial cortex helps the animal switch between competing spatial strategies or competing cue types. Using T-maze alternation, retrosplenial lesion rats were challenged with situations in which the available spatial information between the sample and test phases was changed, so taxing the interaction between different cue types. Clear lesion deficits emerged when intra- and extra-maze cues were placed in conflict (by rotating the maze between the sample and choice phases), or when the animals were tested in the dark in a double-maze. Finally, temporary inactivation of the retrosplenial cortex by muscimol infusions resulted in a striking deficit on standard T-maze alternation, indicating that, over time, other sites may be able to compensate for the loss of the retrosplenial cortex. This pattern of results is consistent with the impoverished use of both allocentric and directional information, exacerbated by an impaired ability to switch between different cue types.

## Introduction

The retrosplenial cortex (areas 29 and 30) comprises the posterior part of the rodent cingulate cortex and is thought to contribute to spatial cognition as well as contextual and episodic memory (Vann et al., 2009; Miller et al., 2014). There are many reasons to suppose that the retrosplenial cortex is important for spatial learning and navigation. Approximately 10% of retrosplenial neurons in the rat brain are ‘head-direction’ cells. That is, the neurons are more active when the rat is facing in a particular direction (Chen et al., 1994; Cho and Sharp, 2001). Furthermore, permanent lesions of the rat retrosplenial cortex can impair location learning in the water-maze. Deficits occur when the escape platform is in a constant position across sessions (Sutherland et al., 1988; Warburton and Aggleton, 1998; Whishaw et al., 2001; Harker and Whishaw, 2002; Vann and Aggleton, 2002; Vann et al., 2003; Lukoyanov et al., 2005) and when the platform position is fixed within a session, but moves between sessions (Sutherland et al., 1988; Vann et al., 2003; Harker and Whishaw, 2004). The retrosplenial cortex is also heavily interconnected with the hippocampus and anterior thalamic nuclei, structures known to be vital for rodent spatial memory (O’Keefe and Nadel, 1978; Sutherland and Rodriguez, 1989; Vann et al., 2009).

Despite the foregoing evidence, there are reasons to suppose that the retrosplenial cortex has a very different role in spatial memory from that of the hippocampus and anterior thalamic nuclei. This potential functional difference is highlighted by the outcome of permanent lesions in these three sites on T-maze alternation. In this task, a rat typically runs up an alley and is then forced into one side arm (‘sample’). After a delay, the rat runs up the alley and is rewarded for alternating, i.e., selecting the opposite arm to that entered in the sample phase (‘choice’). Both hippocampal and anterior thalamic lesions produce very clear deficits, even when the task is made easy by having short retention periods and by extending the inter-trial intervals to reduce interference (Aggleton et al., 1986; Béracochéa and Jaffard, 1994; Aggleton et al., 1996; Bannerman et al., 1999; Dudchenko et al., 2000). In contrast, cytotoxic lesions of the retrosplenial cortex have often failed to impair T-maze alternation, even after extending the retention delay (Neave et al., 1994; Aggleton et al., 1995). One possible explanation for these null results is that the retrosplenial cortex lesions had spared the most caudal parts of the region (Aggleton and Vann, 2004). In response, more complete, retrosplenial cortex lesions were tested, but still only produced borderline T-maze alternation acquisition deficits (Pothuizen et al., 2008). This result remains strikingly different to the severe impairments associated with either hippocampal or anterior thalamic damage.

The present study examined several different explanations for the seemingly mild effects of retrosplenial cortex lesions. One explanation centers on the spatial demands of the task. Alternation in a T-maze could be based on multiple cues types, e.g., allocentric, intra-maze, and directional (Dember and Fowler, 1958; Dudchenko, 2001; Futter and Aggleton, 2006). If retrosplenial cortex lesions only disrupt one cue type, the rat is still potentially able to perform the task effectively by using remaining cue types. Preliminary support for this explanation comes from evidence that the effects of retrosplenial cortex damage on T-maze alteration were only clearly exposed when the rats were thought to rely on directional alternation (Pothuizen et al., 2008), a strategy that might tax head-direction information. By the same reasoning, hippocampal lesions and anterior thalamic lesions must either disrupt multiple cue categories or make it very difficult for the rats to rely on any remaining spatial information (Aggleton and Nelson, 2015). One set of studies (Experiments 1 and 2), therefore, sought to limit the strategies available for T-maze alternation, and so determine whether the retrosplenial cortex is important for just one cue type.

A second explanation arises from the hypothesis that the retrosplenial cortex helps the animal switch between competing strategies or competing cue types (Cooper and Mizumori, 1999; Byrne et al., 2007; Vann et al., 2009). In normal T-maze alternation the rat is able to persist with a fixed strategy, using the same sets of spatial cues at the choice point. This situation contrasts with that in the water-maze, where rats typically start each trial from a new location with a new perspective. Consequently, Experiments 1 and 2 also examined situations when rats switched spatial information types between the sample and choice phases of the T-maze alternation task.

A third explanation (Experiment 3) is that other sites, e.g., the hippocampus and anterior thalamic nuclei, are able to compensate, over time, for loss of the retrosplenial cortex. Such a mechanism would help explain the mild deficits associated with permanent retrosplenial lesions. For this reason, we sought to inactivate the retrosplenial cortex temporarily by infusing the GABA_A_ agonist muscimol. Previous studies that have made transient retrosplenial lesions by infusing tetracaine suggest that this approach might be more disruptive to spatial learning than permanent lesions (Cooper and Mizumori, 1999, 2001).

## Materials and Methods

### Subjects

Subjects were male rats (Lister-Hooded strain; Harlan Bicester, UK) weighing 222–262 grams at the time of surgery. Animals were housed in pairs under diurnal light conditions (14 h light/10 h dark) and testing was carried out during the light phase. Animals were given free access to water throughout the study. The animals were handled daily for at least 1 week before surgery. In Experiments 1 and 2 the rats were randomly assigned to one of two surgical groups: receiving ‘complete’ retrosplenial cortex lesions (‘RSC’) or sham operations (‘Sham’). A total of 15 (Experiment 1) and 14 (Experiment 2) rats received retrosplenial lesions, while 11 (Experiment 1) and 12 (Experiment 2) rats received sham surgeries. Separate cohorts of rats were used in Experiments 1 and 2. In Experiment 3, 12 new rats were implanted with guide cannulae into the retrosplenial cortex. Of these 12, eight were tested on the T-maze task and the remaining four used to image the spread of muscimol. All experiments were carried out in accordance with UK Animals (Scientific Procedures) Act, 1986 and EU directive 2010/63/EU.

#### Surgery Neurotoxic Retrosplenial Cortex Lesions (Experiments 1 and 2).

Animals were deeply anesthetized by an intraperitoneal (i.p.) injection (60 mg/kg) of 6% sodium pentobarbital (Sigma Chemical Company Ltd, Poole, UK, freshly dissolved in saline). Anesthesia was maintained with isoflurane (0.5% in 0_2_). The rats were each placed in a stereotaxic headholder (David Kopf Instruments, Tujunga, CA, USA) with the nose bar at +5.0. The scalp was then cut and retracted to expose the skull. A craniotomy exposed the dura above the dorsal cortex. The lesions were made by injecting a solution of 0.09M *N*-methyl-D-aspartic acid (NMDA; Sigma) dissolved in phosphate buffer (pH 7.2).

Retrosplenial cortex lesions were made by injecting seven sites per hemisphere using a 1 μl Hamilton syringe (Bonaduz, Switzerland) at an infusion rate of 0.1 μl/min. The needle was left in place for 5 min after each infusion. The anterior–posterior (AP) coordinates were measured (in mm) with respect to bregma, the medio-lateral (ML) coordinates (in mm) with respect to the sagittal sinus, and the dorso-ventral (DV) coordinates (in mm) with respect to the surface of the cortex. The stereotaxic co-ordinates of the seven injections were: (1) 0.26 μl at -1.8 (AP), ±0.4 (ML), -1.0 (DV); (2) 0.26 μl at -2.8 (AP), ±0.4 (ML), -1.1 (DV); (3) 0.26 μl at -4.0 (AP), ±0.4 (ML), -1.1 (DV); (4) 0.26 μl at -5.3 (AP), ±0.4 (ML), -2.4 (DV); (5) 0.26 μl at -5.3 (AP), ±0.9 (ML), -1.4 (DV); (6) 0.26 μl at -5.3 (AP), ±0.9 (ML), -1.8 (DV); (7) 0.1 μl at -7.5 (AP), ±1.0 (ML), -1.1 (DV).

On completion of the surgery, the skin was sutured and Clindamycin anti-biotic powder (Pfizer, Walton Oaks, UK) was applied topically. Subcutaneous Metecam (0.03 ml of a 5 mg/ml solution, Buehringer Ingelheim Lid, Bracknell, UK) provided peri-operative analgesia. Animals also received subcutaneous injections of glucose saline (5 ml). The surgical operated controls (‘Sham’) received the same procedure and drugs as the lesioned animals, with the exception of lowering the needle into the brain and injection of NMDA. Following surgery, rats were allowed a minimum of 2-weeks of post-operative recovery before behavioral testing.

#### Cannulae Implantation into the Retrosplenial Cortex (Experiment 3)

Anesthesia was induced by isoflurane (4%) in O_2_ and maintained thereafter with isoflurane (1–2%). Stereotaxic surgery was conducted with the incisor bar set at -3.3 mm below the intra-aural line, so that the skull was flat. Given the rostral-caudal extent of the retrosplenial cortex, the rats were implanted with two bilateral guide cannulae: one targeting the rostral (approximately -2.5 posterior to bregma) and a second targeting the caudal (approximately -6.0 posterior to bregma) retrosplenial cortex. At approximately -2.5 (AP) from bregma a small flap of bone was removed to reveal the central sinus. Medial lateral coordinates were then taken with reference to the central sinus. At the rostral site, one bilateral stainless guide cannulae (26 gage, with 1.5 mm projection below the guide; Plastic One, Roanoke, VA, USA) was implanted ±0.7 from the central sinus. At the caudal site, two holes were drilled ±1.0 from the central sinus and a second bilateral stainless guide cannula (26 gage, with 1.7 mm projection below the guide; Plastic One, Roanoke, VA, USA) was implanted. Cannulae were held in place by dental cement and anchored to the skull with six fixing screws (Bilaney Consultants Ltd, Sevenoaks, UK) located on the parietal bone plates. Removable obturators (Plastic One, Roanoke, VA, USA) were inserted into the guide cannulae to prevent the cannulae from blocking.

All other aspects of the surgical procedures were the same as described for Experiments 1 and 2, except that the rats were allowed 1 week of post-operative recovery before behavioral testing.

### Apparatus

In Experiment 1 and 3, as well as stages 1–2 of Experiment 2, testing was conducted in a modifiable four-arm (cross-shaped) maze. The four arms (70 cm long, 10 cm wide) were made of wood while the walls (17 cm high) were made of clear Perspex. At any time, one of the arms could be blocked off to form a T-shaped maze. Aluminum barriers could be positioned ∼25 cm from the end of each arm to create a start area. For all experiments, the location of the start arm remained constant such that the T-maze was in the same orientation throughout testing. The maze, which was supported by two stands (94 cm high), was situated in a rectangular room (280 cm × 280 cm× 210 cm) with salient visual cues located on the walls.

In stage 3 of Experiment 2, two identical cross-mazes were used. The mazes were placed side-by-side (with the end of the two side arms touching each other) on a table (74 cm high) in the same experimental room. The floor of each cross-maze was made from wood and painted white. Each arm was 45.5 cm long and 12.0 cm wide. The sidewalls (32.5 cm high) were made from opaque black Perspex. At the end of each arm was a sunken food well (2 cm in diameter and 0.75 cm deep). Access to an arm could be prevented by placing an aluminum barrier at the entrance of the arm.

Lighting during initial habituation and training in the ‘light’ stages was provided by two standard ceiling lights, giving a mean light intensity of 140.8 lx (measured at the center of the maze). During training in the ‘dark’ stages, illumination was provided by two infra-red spotlights, with the experimenter using night-vision goggles (Productive Firm Dipol Ltd.) in order to see. Mean light intensity within the T-maze during training in the dark was less than 1 lx.

### Procedure

One week prior to the start of the experiment, the animals were placed on a food restricted diet and gradually reduced to 85% of their free-feeding weights. One day before the start of the experiment, the rats were familiarized with the sucrose reward pellets (45 mg; Noyes Purified Rodent Diet, UK) in their home cage.

#### Pre-Training

Pre-training began with 4 days of habituation to the maze. On Day 1, four sucrose pellets were scattered down each of the arms and two in each food well, and the rats were allowed to explore the maze for 5 min. On Day 1 only, rats were habituated in cage pairs. On Days 2 and 3, the number of pellets was increased to four in each well and the rats were habituated to the maze for 5 min. On Day 4, the number of pellets was reduced to two in each well and the aluminum barrier was placed at the entrance of one of the arms. The rats were again allowed to explore the maze for 5 min. During each habituation session, the reward pellets were continuously replaced so that no arm was found to be empty on return. Training on the forced-choice alternation rule began the following day.

#### Basic Experimental Procedure for All Three Experiments

**FIGURE 1:**
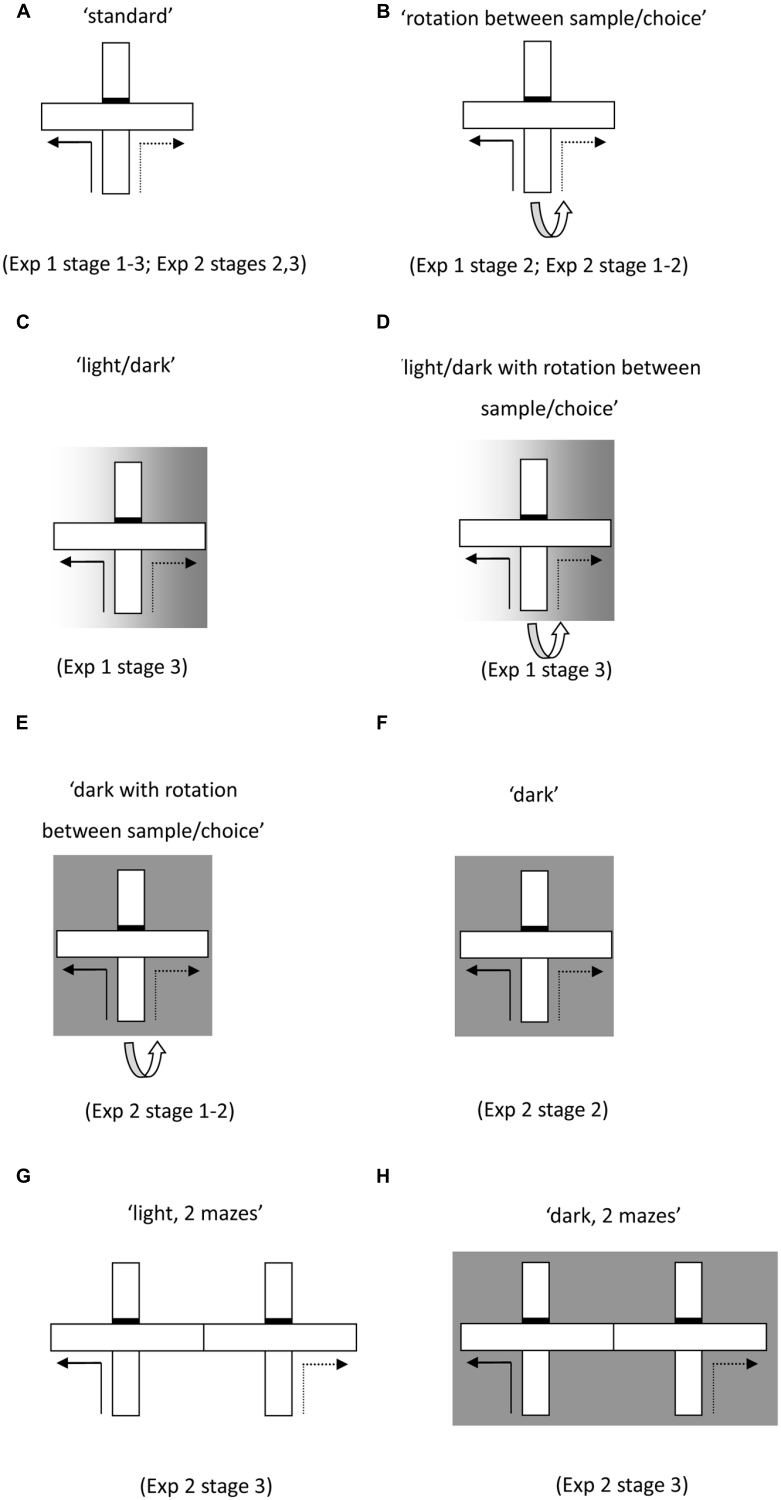
**Schematics of test protocols used for the forced-choice alternation task run in a single maze **(A–F)** and then in two adjacent mazes (G,H)**. Solid lines indicate the forced sample phase and the dashed lines indicate the correct response in the choice phase. Initial training **(A)** permitted the use of multiple cue types. The task was then modified to nullify the value of certain cue types. These manipulations included rotating the maze between the sample and choice phase **(B,D,E)**, running the sample phase in the light and the choice phase in the dark **(C,D)**, as well as running both phases in the dark **(F,H)**.

Throughout Experiment 1, the rats received six trials per daily session. In Experiments 2 and 3 this was increased to eight trials. Each trial consisted of a forced ‘sample’ run followed by a ‘choice’ run (**Figure 1**). Although all testing was carried out in a cross-maze, it was modified by placing a barrier blocking access to the arm directly in line with the start arm (effectively turning it into a T-maze configuration).

During the forced sample run, one of the side arms of the maze was blocked by an aluminum barrier. After the rat turned into the preselected arm, it was allowed to eat two reward pellets that had been previously placed in the food well. The rat was then picked up from the maze and immediately returned to the start arm. Approximately 10 s after the end of the sample phase, the choice phase began. The rat was allowed to run up the stem of the maze and was now given a free choice between the left and the right turn arms (**Figure 1A**). The rat received two reward pellets only if it turned in the direction opposite to that in the sample run (i.e., non-matching). The choice of the sample arm (left or right) was randomly assigned with the only stipulation that no arm could be selected as the sample on more than two consecutive trials. In Stage 3 of Experiment 2 (performed in the two adjacent cross-mazes) the sample phase was conducted in one cross-maze and the choice phase in the adjacent cross-maze.

At the start of each session, rats were taken from the holding room to the experimental room in an enclosed, opaque carry-box made of aluminum. Each rat was placed in a separate compartment in the box. Groups of either three or four rats were tested together, with each rat having one trial in turn, so that the inter-trial interval was ∼3 min.

Experiments 1 and 2 examined whether the involvement of the retrosplenial cortex in alternation problems is restricted to certain classes of cues. In order to control systematically the strategies supporting alternation, the alternation training consisted of different stages and trial types that allowed the availability of different cues to be selectively manipulated (see **Figure 1**).

### Experiment 1

#### Stage 1 (All Cue Types Available)

In Stage 1 (six sessions), both the sample and choice run were carried out in the light in the single maze, with no rotation of the maze between sample and choice (see **Figures 1A**, ‘standard’).

#### Stage 2 (Intra-Maze Cues in Conflict)

In order to place intra-maze cues in conflict, the maze was rotated 90° between the sample and choice phases. Once the sample phase was completed, the rat was removed from the sample arm and the start arm was rotated 90° (**Figure 1B**. ‘rotation between sample/choice’). In order to prevent the rats from acquiring a rule, the direction of rotation (either clockwise or anti-clockwise) was randomly selected so that on half the trials it was rotated clockwise and on the other half it was rotated anticlockwise (there were never more than two successive rotations in the same direction). There were two sessions (six trials a session) of standard trials followed by 4 days of rotation between the sample and choice phases, then finally 2 days of standard trials.

#### Stage 3 (Intra-Trial Switch in Lighting Conditions)

In order to probe the flexible use of the various cues available to the rats, the illumination in the room was changed between the sample and choice phases in some trials of Stage 3. The sample phase was conducted in the light but once the sample phase was completed, the lights in the room were switched off to preclude the use of distal visual cues (**Figure 1C**, ‘light/dark’). There were 10 days of training with six trials per day (three standard trials in the light, three trials with the change in lighting conditions between the sample and choice phases). There then followed 2 days (six trials a day) in which both the maze was rotated and the lighting was switched off between the sample and choice phases (**Figure 1D**, ‘light/dark with rotation’).

### Experiment 2

#### Stage 1 (Intra-Maze Cues in Conflict in the Light and Dark)

The rats had 16 days (eight trials a day) in the single T-maze. Half of the trials on each day were conducted in the light, with the maze rotated between sample and choice (**Figure 1B**, ‘rotation between sample/choice’). To exclude the use of allocentric cues, the remaining trials were conducted in the dark, with the maze again rotated between sample and choice (**Figure 1E**, ‘dark with rotation between sample/choice’).

#### Stage 2 (Intra-Maze Cues in Conflict in the Light and Dark Plus Standard Trials in the Light and Dark)

In order to determine whether the within session switch in trial type may have contributed to the pattern of results obtained, the animals underwent a further 14 days of training (eight trials a day) in which the trial type remained constant within each session. Rats had 4 days in the light without rotation (**Figure 1A**, ‘standard’), 2 days in the light with rotation (**Figure 1B**, ‘rotation between sample/choice’), 4 days in the dark without rotation (**Figure 1F**, ‘dark’), and finally 2 days in the dark with rotation (**Figure 1E**, ‘dark with rotation between sample/choice’).

#### Stage 3 (Absence of Intra-Maze Cues, Allocentric Cues Ambiguous, in Light and Dark)

The final stage was conducted in two adjacent T-mazes, so that any intra-maze cues were uninformative. Initially, the rats had 2 days (1 day in each maze) in a single maze with the sample and choice phases conducted in the same maze. Thereafter, the rats had 8 days (eight trials a day) in the two mazes conducted in the light (**Figures 1G**, ‘light two mazes’). The sample phase was conducted in one maze and the choice phase in the adjacent maze. There followed 8 days of the same training except that it was conducted in the dark (**Figure 1H**, ‘dark two mazes’).

### Experiment 3

#### Pre-Surgical Training

In Experiment 3, pre-training and initial task acquisition were conducted prior to surgery. Following 4 days of pre-training (as described for Experiments 1 and 2), the rats underwent 6 days of training on the standard T-maze task, receiving eight trials a day. This training was conducted in exactly the same manner as described for Stage 1 training in Experiments 1 and 2 and in the same adapted cross-maze.

#### Post-Surgical Training

Following recovery from surgery, the rats were given 1 day of reminder training on the standard T-maze task. This training proceeded in exactly same manner as the pre-surgery training.

#### Muscimol Inactivation of the Retrosplenial Cortex

The animals underwent two test sessions. Prior to these test sessions, the animals received either infusions of muscimol or phosphate buffered saline (PBS). Half the animals were tested first under muscimol and then tested after the control infusion. For the other half of the animals, the test order was reversed. An interval of 48 h separated each test.

Muscimol (Sigma, Poole, UK) was dissolved in sterile PBS at a dose of 1 mg/ml (Holt and Maren, 1999). Rats were lightly restrained, the obturators removed, and 33-gage stainless steel infusion cannulae (Plastic One, Roanoke, VA, USA) that projected 1.5 mm beyond the tip of the guide cannulae were inserted bilaterally into both the rostral and posterior retrosplenial cortex. Each pair of infusion cannulae were connected to two 5 μl Hamilton syringes (Bonaduz, Switzerland) mounted on two infusions pumps (Harvard Apparatus Ltd, Kent, UK). A volume of 0.2 μl per infusion site (four in total) was infused over 1 min. The infusion cannulae were left *in situ* for a further 1 min to allow absorption of the bolus. The infusion cannulae were then removed and the obturators replaced. The animals were returned to their home cage. After 30 min, the animals received eight standard T-maze trials.

### Histology

#### Histological Evaluation of the Lesions and Cannulae Placement

The boundaries and nomenclature for the retrosplenial cortex are taken from van Groen and Wyss (1992). Histological procedures included the staining of coronal sections for Nissl substance. At the end of behavioral testing, the rats were deeply anesthetized with sodium pentobarbital (60 mg/kg, i.p, Euthatal, Merial Animal Health) and then transcardially perfused with 0.1 M PBS at room temperature for ∼2 min (flow rate 35 mL/min), followed by a 4% solution of depolymerized paraformaldehyde in 0.1 M phosphate buffer for ∼10 min at a flow rate of 35 mL/min. The brains were removed and post-fixed for 4 h in the same fixative and then cyroprotected in 25% sucrose solution (in PBS) overnight. Four adjacent series of coronal sections (40 μm) were cut on a freezing sliding microtome. Three series were collected and stored in cyroprotectant for subsequent processing. One in four series was directly mounted onto gelatin-coated slides and, when dry, stained with cresyl violet. The sections were then dehydrated through an alcohol series, cleared with xylene, and cover-slipped with the mounting medium DPX.

#### Imaging Muscimol Spread

In order to image the spread of muscimol around the injection site, four rats were infused with fluorophore-conjugated muscimol (Life Technologies, Paisley, UK) into the retrosplenial cortex (Allen et al., 2008). The infusion procedure was identical to the one for the standard muscimol infusions described above. The conjugated muscimol was dissolved in sterile PBS to a dose of 1mg/ml. The molecular weight of conjugated muscimol is approximately six times larger than the molecular weight of standard muscimol (Allen et al., 2008) and consequently its spread is likely to be more restricted than that of standard muscimol. For this reason, and to give a conservative estimate of the spread of standard muscimol, we infused two rats with a volume of 0.6 μl of conjugated muscimol (three times the volume of muscimol used in Experiment 3). A further two rats were infused with a volume of 0.2 μl of conjugated muscimol. Thirty minutes after the infusions, the rats were deeply anesthetized with sodium pentobarbital (60 mg/kg, i.p, Euthatal, Merial Animal Health). All other histological procedures were as described before, except that the sections were not stained for Nissl.

### Statistical Analyses

The number of correct responses per session and per trial type was noted. The percentage correct per trial type was then calculated and averaged across two sessions to give blocks of 12 (Experiment 1) and 16 (Experiment 2) trials. This measure was subjected to ANOVA with between subject factor group (RSC and Sham) and within subject factors of trial type and block. In Experiment 3, test performance was subjected to ANOVA with a within subject factor of infusion (muscimol and control). To confirm that the assumptions of ANOVA were met, the data were subjected to Levene’s Test of Equality of Variance, the Shapiro–Wilk Test of Normality and, where appropriate, Mauchely’s Test of Sphericity. The alpha level was set at *p* < 0.05. Simple effects were used to explore statistically significant interactions. To assess whether performance remained above chance (i.e., >50%) one sample *t*-tests were applied to the data, where appropriate.

## Results

### Histological Evaluation of the Lesions

**FIGURE 2:**
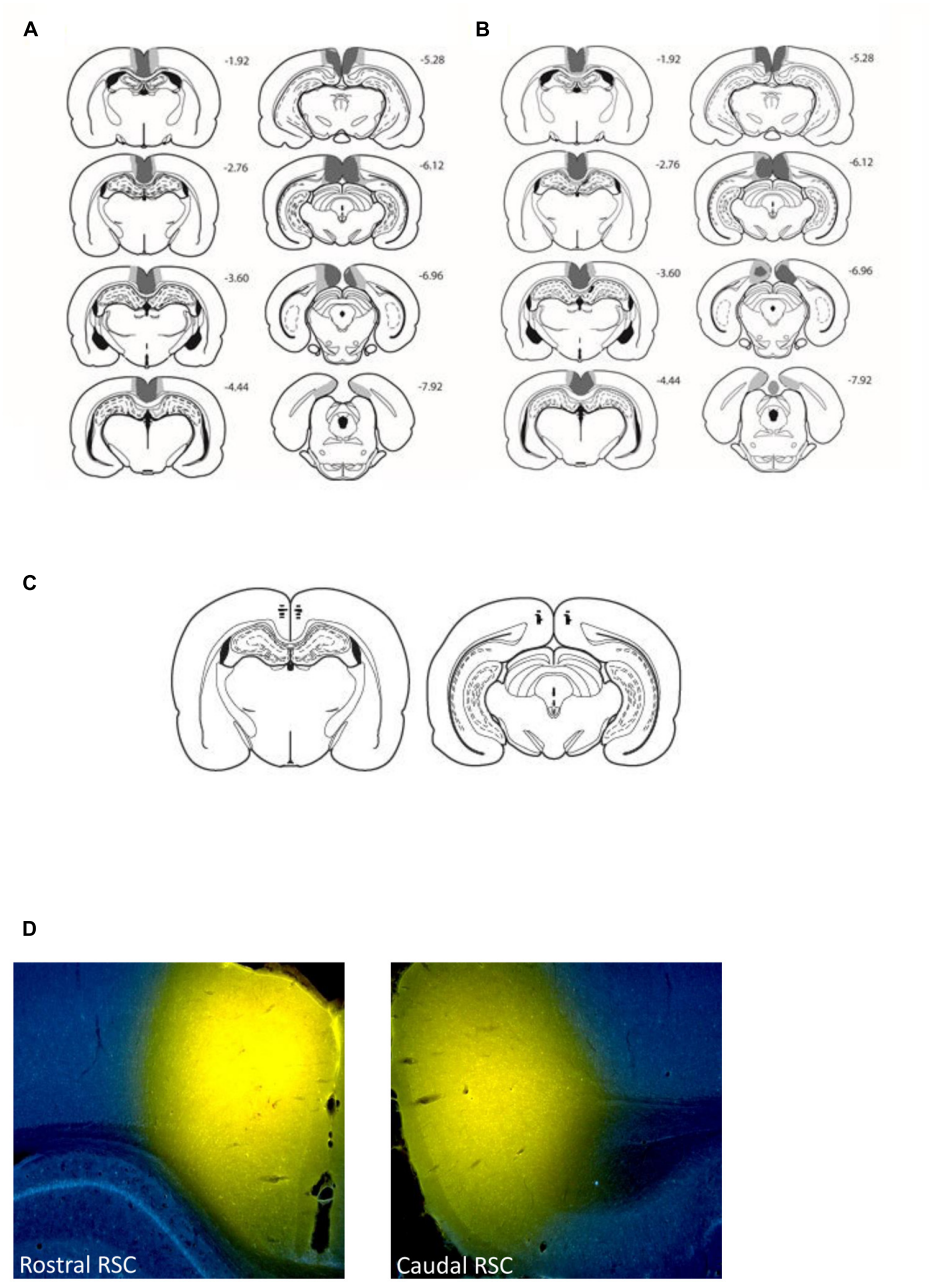
**Location and extent of the retrosplenial cortex lesions (Experiments 1 and 2) and retrosplenial cortex cannulae placements (Experiment 3). The figure depicts the case with the largest (pale gray) and smallest (dark gray) cortical lesions for Experiment 1 (A)** and Experiment 2 **(B)** on a series of coronal sections. The numbers refer to the approximate distance in mm of each section caudal to bregma (Paxinos and Watson, 2005). **(C)** Depicts cannulae placements within the rostral and caudal retrosplenial cortex. **(D)** Depicts the spread of 0.6 μl conjugated muscimol infused in both the rostral and caudal retrosplenial cortex.

A total of seven rats (three from Experiment 1 and four from Experiment 2) were excluded from all subsequent analysis, as these cases had either excessive sparing throughout the retrosplenial cortex or extensive bilateral hippocampal and subicular damage. In the remaining cases (12 in Experiment 1 and 10 in Experiment 2), the surgeries consistently produced marked cell loss throughout almost the entire retrosplenial cortex (**Figure 2**). In these cases, much of the cortical tissue had completely collapsed, but in other areas where tissue was still intact, the remaining cells looked abnormal and there was extensive gliosis. Anterior to the splenium, the lesions were essentially complete. Caudal to the splenium, there was partial sparing of granular a retrosplenial cortex in six cases (four bilateral, Experiment 1) and in one case there was bilateral dysgranular sparing. Additional cell loss occurred in a discrete part of the most dorsal medial portion of CA1 in the septal hippocampus (six cases bilateral, Experiment 1; four cases Experiment 2), seven cases unilateral (three cases Experiment 1, four cases Experiment 2). There was evidence of ventricular dilation in five cases (Experiment 1).

### Histological Evaluation of the Muscimol Infusions

**Figures 2C** displays the location of the tips of the cannulae in the retrosplenial cortex from the eight animals that performed the T-maze task. In all eight animals the cannulae tips were located bilaterally in both the caudal and rostral retrosplenial cortex. Examination of the four additional cases that received infusions of conjugated muscimol demonstrated that the spread of both 0.2 and 0.6 μl infusions was restricted to the retrosplenial cortex. Consistent with previous reports (Allen et al., 2008) white matter appeared to acts as a diffusion barrier such that the rostral infusions did not diffuse through the corpus callosum into the dorsal hippocampus. **Figures 2D** displays the diffusion gradients in both rostral and caudal retrosplenial cortex of an animal infused with 0.6 μl of conjugated muscimol.

### Behavioral Results

#### Experiment 1

##### Stage 1 – standard spatial alternation

**FIGURE 3:**
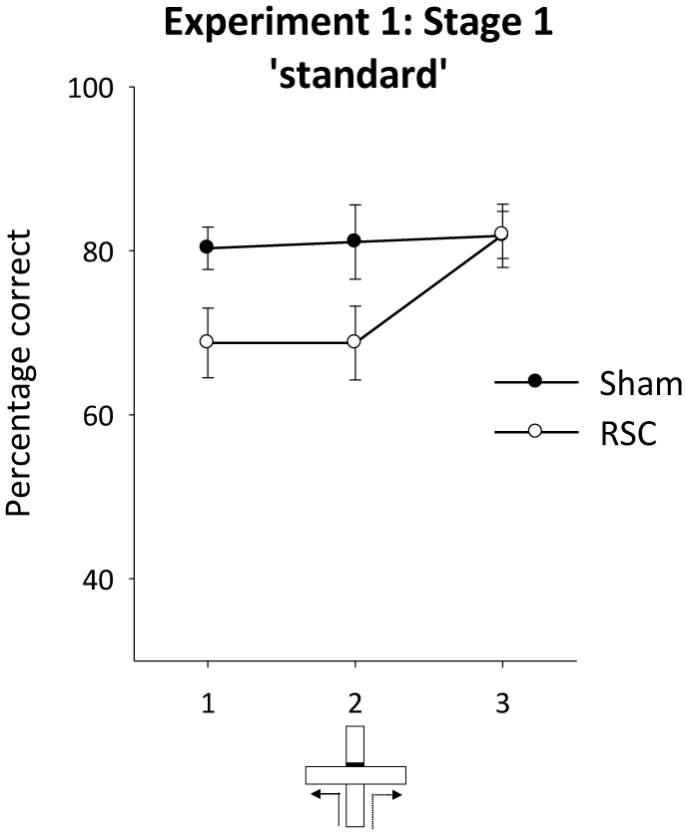
**Experiment 1, stage 1**. Acquisition of the forced-choice alternation rule. The graphs depict the mean percentage correct responses across three blocks of sessions (12 trials per block). Error bars refer to ±SEM. RSC, retrosplenial cortex lesions; Sham, surgical controls.

**Figure 3** displays the performance of both groups across the three blocks of two sessions of training on the standard alternation task (**Figure 1A**). As is clear from this figure, the RSC group were initially mildly impaired relative to the Sham group, but by the end of training there were no differences between the groups. ANOVA revealed an effect of lesion [*F*_(1,21)_ = 4.4, *p* < 0.05] but no effect of block or interaction between lesion and block [maximum *F*_(2,21)_ = 2.99, *p* = 0.06]. Performance in the RSC group was also significantly above chance levels across the three blocks of training [minimum *t*_(11)_ = 4.2, *p* < 0.005].

##### Stage 2 – intra-maze cues in conflict

**FIGURE 4:**
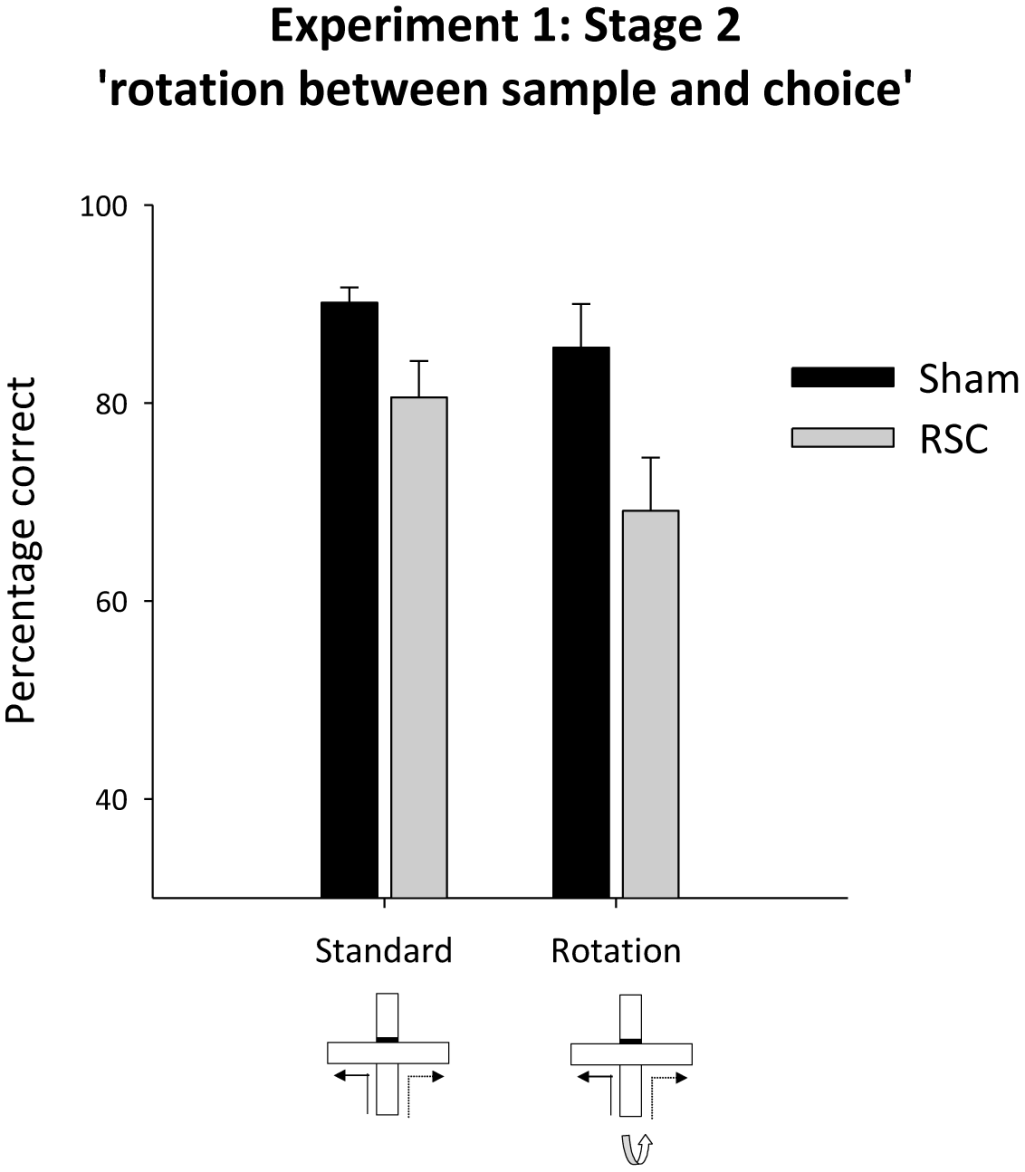
**Experiment 1, stage 2**. Intra-maze cues in conflict. Performance on the forced-choice alternation rule during standard training and when the maze was rotated between the sample and choice phases. Error bars refer to ±SEM. RSC, retrosplenial cortex lesions; Sham, surgical controls.

Placing intra-maze cues in conflict (by rotating the maze between the sample and choice phases, **Figure 1B**) led to a numerical drop in performance in both groups of animals (**Figure 4**). There was no statistical evidence that the two groups were differentially sensitive to maze rotation as there was an effect of rotation [*F*_(1,21)_ = 5.9, *p* < 0.05] and lesion [*F*_(1,21)_ = 7.7, *p* < 0.05] but no rotation by lesion interaction [*F*_(1,21)_ = 1.1, *p* = 0.31].

##### Stage 3 – intra-trial switch from light to dark

**FIGURE 5:**
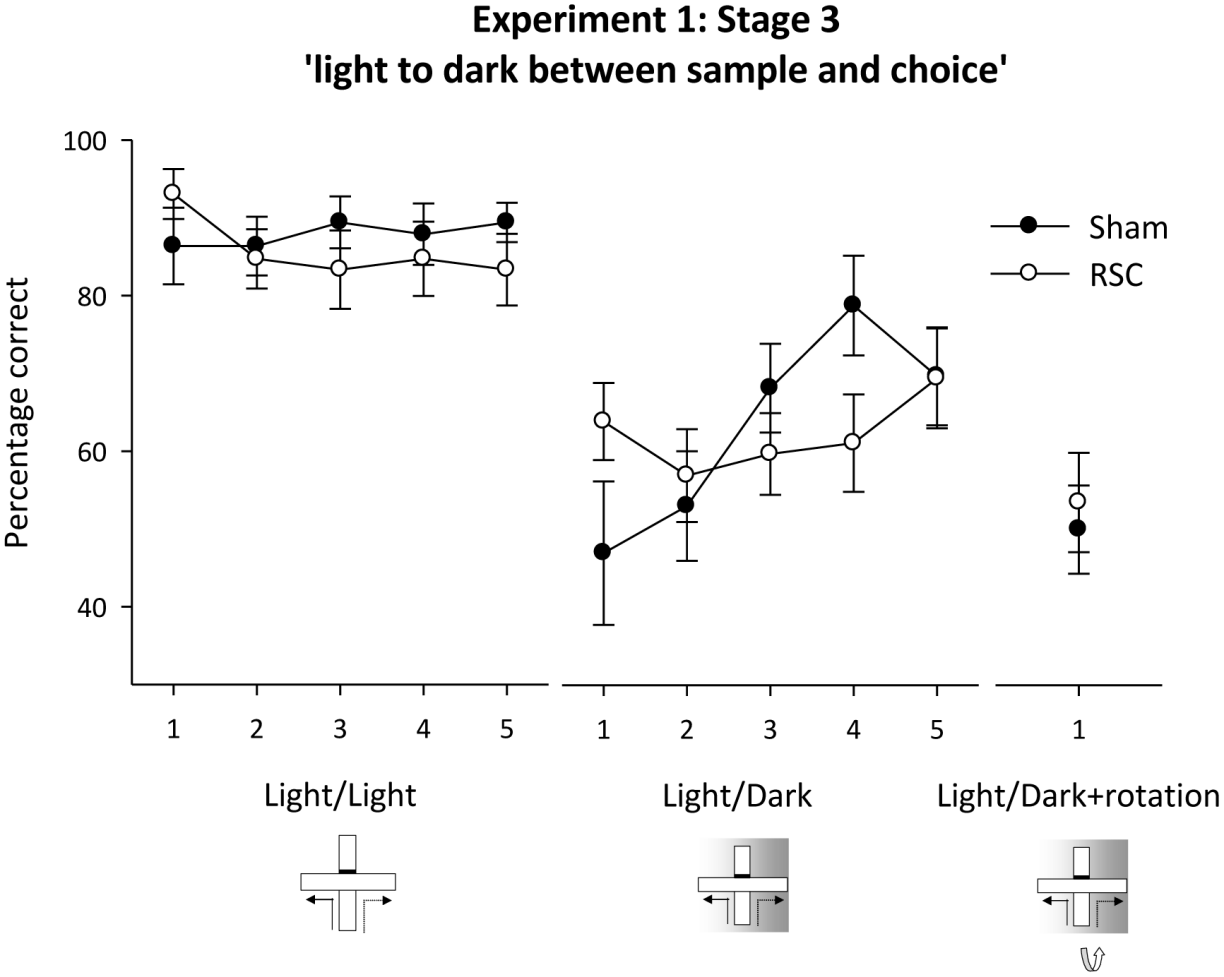
**Experiment 1, stage 3**. Intra-trial change in lighting conditions. Performance on the forced-choice alternation rule when there was a change in the lighting conditions between the sample and choice phases. The line graph shows the mean percentage correct responses in blocks of 12 trials for intermixed trials when either there was no change in lighting between sample and choice (light/light, i.e., standard) or when there was a switch to running in the dark between the sample and choice (light/dark). For the final two sessions, this light to dark switch was combined with maze rotation (light/dark + rotation). Error bars refer to ±SEM. RSC, retrosplenial cortex lesions; Sham, surgical controls.

When all cues types were available (**Figure 1A** – Stage 3, standard), there was no difference in accuracy between the two groups (all *F*s < 1) and performance in both groups was above chance for each of the five training blocks of trials [**Figure 5**; Sham minimum *t*(10) = 7.4, *p* < 0.001; RSC minimum *t*_(11)_ = 6.6, *p* < 0.001].

Initially during the concurrent light/dark trials (see **Figure 1C**), the RSC group appeared less sensitive to the intra-trial change in lighting conditions as performance in Sham animals dropped to around chance levels (**Figure 5** – light/dark). But, as training proceeded, Sham performance recovered whereas the RSC group showed little or no improvement. ANOVA revealed an effect of block [*F*_(4,84)_ = 3.1, *p* < 0.05], a marginal block by lesion interaction [*F*_(4,84)_ = 2.4, *p* = 0.055] and no effect of lesion (*F* < 1). The RSC group showed no improvement in performance across the blocks of training (*F* < 1) but in the Sham group there was an effect of block [*F*_(4,40)_ = 4.5, *p* < 0.01] as accuracy improved across the five blocks of training. Further analysis showed that performance in the Shams was not above chance levels on blocks 1–2 (maximum *t* < 1) but was above chance on blocks 3–5 [minimum *t*_(10)_ = 3.1, *p* < 0.05]. In the RSC group, performance was above chance levels on blocks 1 and 5 [minimum *t*_(12)_ = 2.8, *p* < 0.05] but did not differ from chance on blocks 2–4 [maximum *t*_(12)_ = 1.9, *p* = 0.09].

When the intra-trial change in lighting conditions was combined with maze rotation (**Figure 1D**) neither group performed above chance levels (**Figure 5**). This failure to solve the problem was confirmed by one sample *t*-tests [Sham maximum *t*(10) = 0; RSC maximum *t*_(11)_ = 0.6, *p* = 0.6]. ANOVA confirmed there was no difference between the two groups on these trials (*F* < 1).

#### Experiment 2

##### Stages 1 and 2 – intra-maze cues in conflict in the light or dark

**FIGURE 6:**
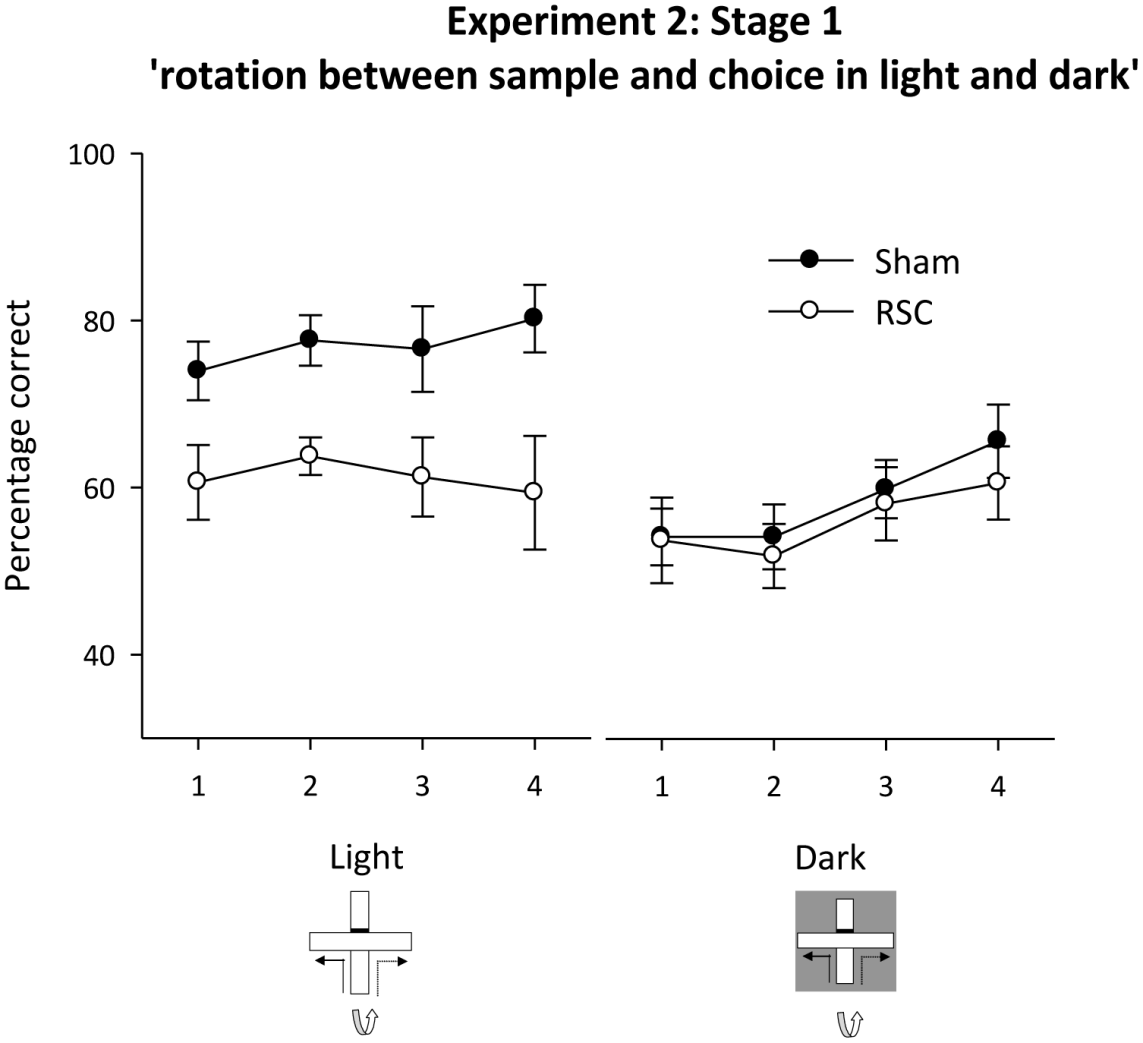
**Experiment 2, stage 1**. Intra-maze cues in conflict in either the light or the dark. Performance on the forced-choice alternation rule when the maze was rotated between the sample and choice phases on all trials. For half of the trials in each session both phases were in the light. For the remaining trials, both phases were in the dark. Error bars refer to ±SEM. RSC, retrosplenial cortex lesions; Sham, surgical controls.

Inspection of **Figure 6** reveals that in the light, placing intra-maze cues in conflict (light + rotation, see **Figure 1B**) appeared to selectively disrupt performance in the RSC group. In contrast, with the absence of visual cues (‘dark with rotation’ see **Figure 1E**), both groups appeared equally sensitive to the manipulation. ANOVA confirmed this description of the data as there was a main effect of lighting [*F*_(1,60)_ = 32.3, *p* < 0.001], lesion [*F*_(1,60)_ = 10.3, *p* < 0.01] but also a lesion by lighting interaction [*F*_(1,20)_ = 11.3, *p* < 0.01] but no other effects or interactions reached significance [maximum *F*_(3,60)_ = 1.7, *p* = 0.2]. The lesion by lighting interaction arose because there was an effect of lesion during light [*F*_(1,20)_ = 26.3, *p* < 0.001] but not dark (*F* < 1) trials.

Subsequent analysis confirmed that Sham performance in the light was consistently above chance [minimum *t*_(11)_ = 3.6, *p* < 0.01] but in the dark it was only above chance on blocks 3–4 [minimum *t*_(11)_ = 2.8, *p* < 0.05, see **Figure 6**]. Performance by the RSC group was above chance in the light on blocks 1–3 [minimum *t*_(9)_ = 2.4, *p* < 0.05] but in the dark it was only above chance on block 4 (**Figure 6**).

**FIGURE 7:**
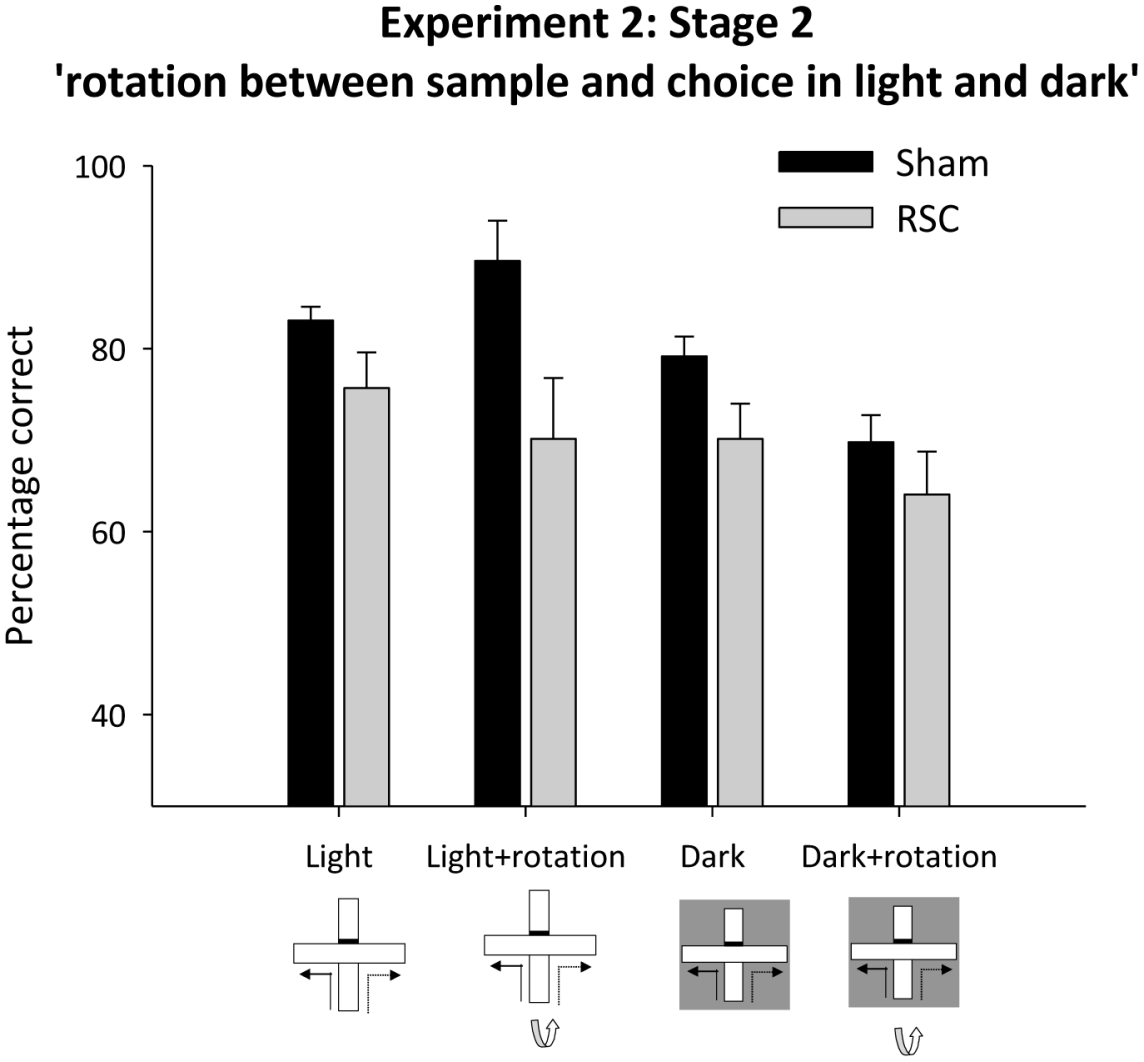
**Experiment 2, stage 2**. Intra-maze cues in conflict in either the light or dark. Performance on the forced-choice alternation rule when the maze was rotated between the sample and choice phases. The bar graph depicts the mean percentage correct responses in the light or dark with and without maze rotation between sample and choice phases. Error bars refer to ±SEM. RSC, retrosplenial cortex lesions; Sham, surgical controls.

In Stage 2 there were four trials types (standard, light + rotation, dark, dark + rotation) but trial type remained constant across each session (see **Figures 1A,B,E,F**). As before, rotation in the light, but not in the dark, produced a lesion deficit (**Figure 7**). ANOVA yielded an effect of lighting [*F*_(1,20)_ = 14.5, *p* < 0.001], a marginal effect of rotation [*F*_(1,20)_ = 4.3, *p* = 0.051], an effect of lesion [*F*_(1,20)_ = 11.3, *p* < 0.01] and a marginal lesion by lighting by rotation interaction [*F*_(1,20)_ = 4.3, *p* = 0.051], but no other interactions [maximum *F*_(1,20)_ = 2.8, *p* = 0.1]. Subsequent simple effects analysis confirmed that there was an effect of lesion in the light + rotation condition [*F*_(1,20)_ = 14.6, *p* < 0.001] but not in the light without rotation condition [*F*_(1,20)_ = 2.9, *p* = 0.1]. Conversely in the dark, Shams were more accurate than the RSC group [*F*_(1,20)_ = 4.4, *p* < 0.05], but when the intra-maze cues were placed in conflict both groups performed at equivalent levels [*F*_(1,20)_ = 1.1, *p* = 0.3]. Further analyses revealed that performance by both the Sham [minimum *t*_(11)_ = 6.7, *p* < 0.001] and the RSC group [min *t*_(9)_ = 3.5, *p* < 0.01] was above chance for each of the four trial types.

##### Stage 3 – intra-maze cues unavailable, visual cues unavailable in the dark (two mazes)

During pre-training (two sessions) in the adjacent cross-mazes (sample and choice phases run in the same maze) there was no difference in accuracy between the two lesion groups [maximum *F*_(1,20)_ = 2.5, *p* = 0.13]. Mean percentage correct (±SEM): Sham = 86.4 (±3.3); RSC = 83.3 (±3.7).

**FIGURE 8:**
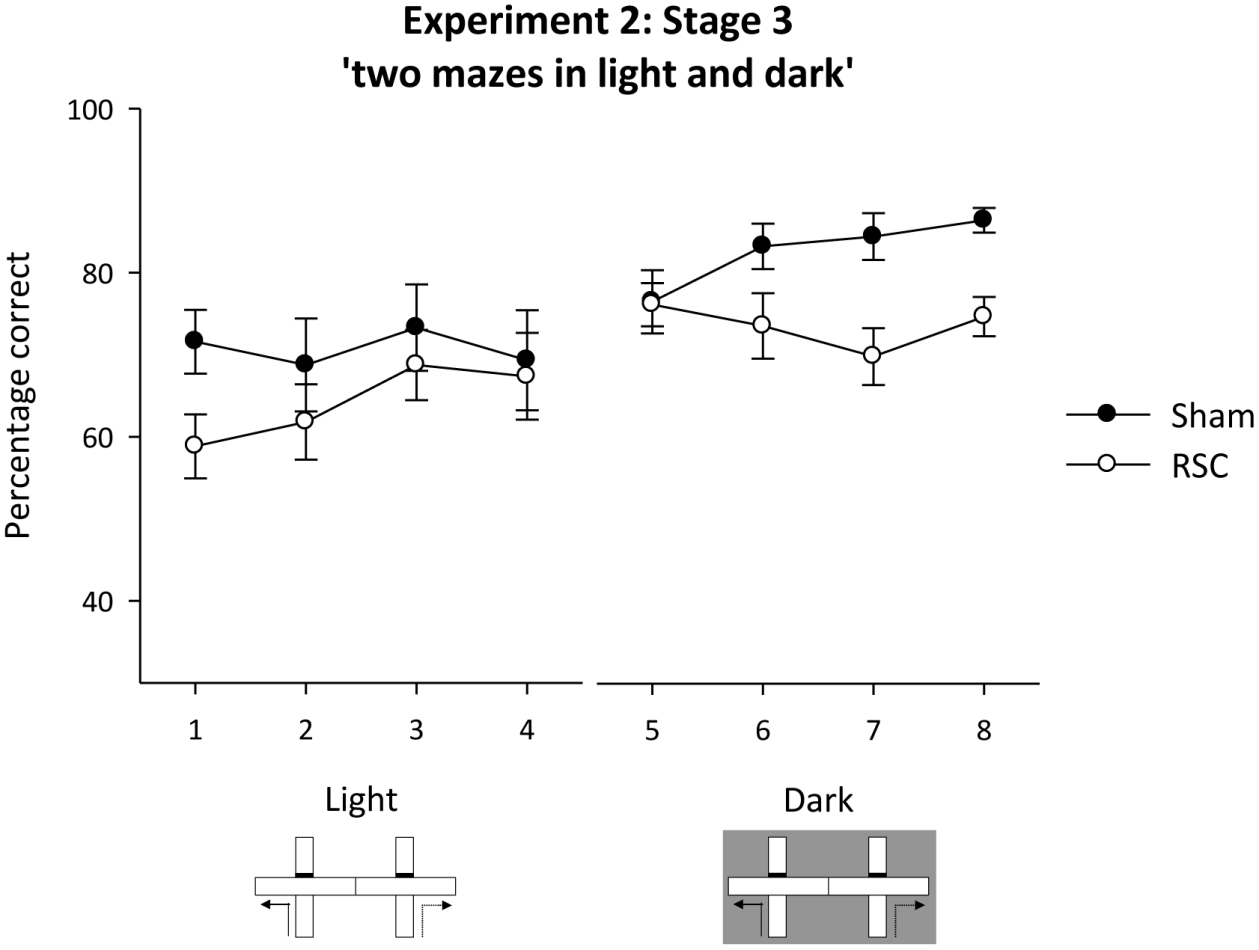
**Experiment 3, stage 3**. Two adjacent mazes to prevent use of intra-maze cues to solve the forced-choice alternation rule. The line graph depicts the mean percentage correct responses in blocks of 16 trials when first tested in the light, then in the dark. Error bars refer to ±SEM. RSC, retrosplenial cortex lesions; Sham, surgical controls.

To exclude the use of intra-maze cues, the sample and choice phases were thereafter run in separate mazes. The animals were tested in the light (**Figure 1G**) and in the dark (**Figure 1H**). In the light, the two groups performed at equivalent levels, but in the dark, the Shams outperformed the RSC group (**Figure 8**). ANOVA revealed a block by lighting by lesion interaction [*F*_(3,60)_ = 3.2, *p* < 0.05], an effect of lesion [*F*_(1,20)_ = 4.5, *p* < 0.05] but no other effects or interactions [maximum *F*_(1,20)_ = 1.9, *p* = 0.17]. The three-way interaction arose because in the light there were no effects of lesion or interaction between lesion and block [maximum *F*_(1,20)_ = 1.4, *p* = 0.24]. However, in the dark, there was a lesion by block interaction [*F*_(3,60)_ = 2.82, *p* < 0.05] as Sham, but not RSC, performance improved across the four blocks of training. Overall, the Shams were more accurate than the RSC group in the dark [*F*_(1,20)_ = 8.7, *p* < 0.01].

#### Experiment 3

##### Pre-surgical training

All animals readily acquired the alternation problem. Performance improved across the three blocks of two sessions, as confirmed by an effect of Block [*F*_(2,14)_ = 5.9, *p* < 0.05] and was consistently above chance levels across all three blocks [minimum *t*_(7)_ = 8.3, *p* < 0.001].

##### Post-surgical training

**FIGURE 9:**
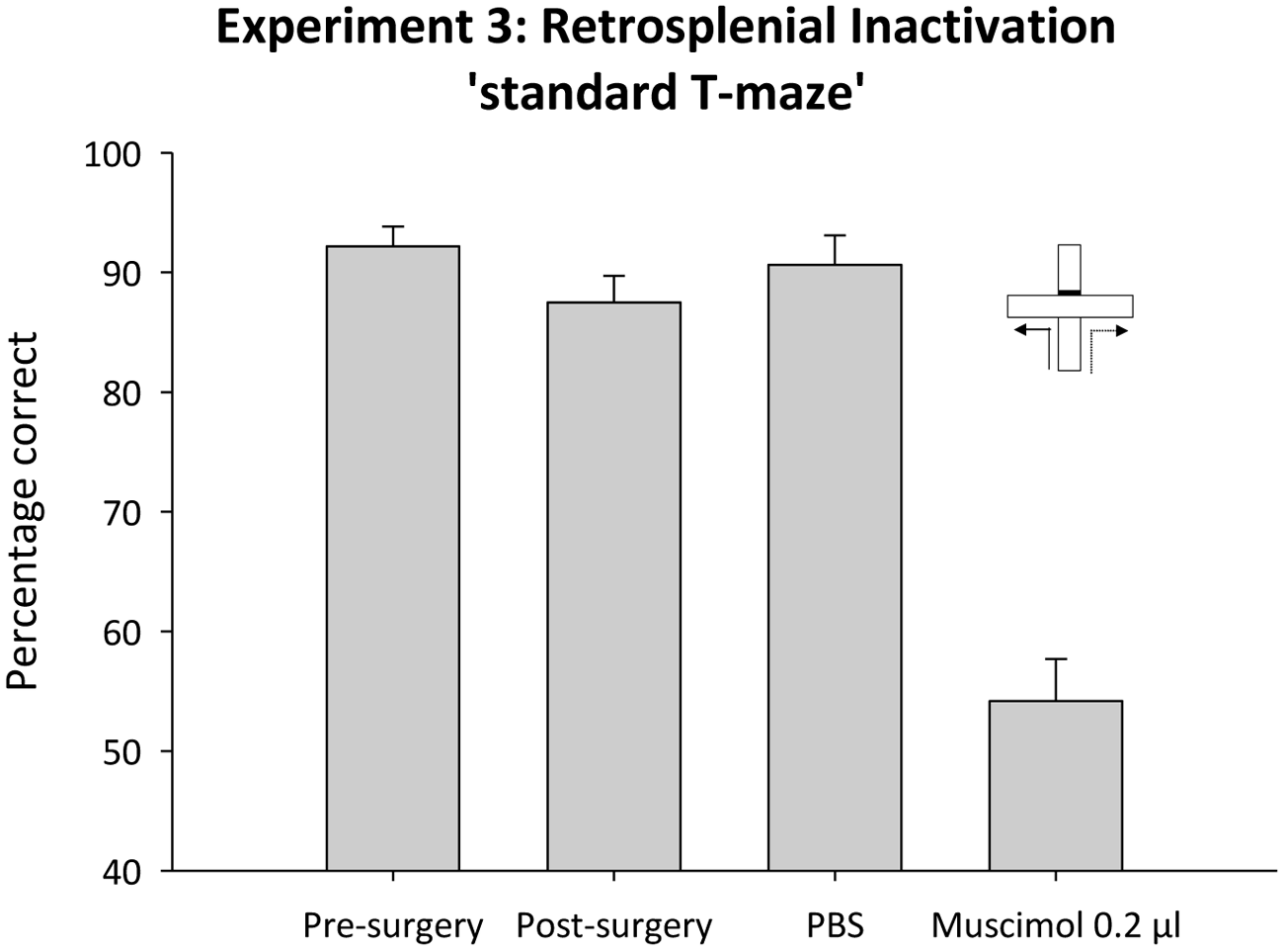
**Experiment 3**. Standard T-maze alternation and retrosplenial cortex inactivation. The bar graph depicts the mean percentage correct responses across the six sessions of acquisition (pre-surgery) and performance in subsequent single sessions (post-surgery, control infusion and 0.2 μl muscimol infusion). Error bars refer to ±SEM.

Following recovery from surgery, performance was again above chance [*t*_(7)_ = 16.8, *p* < 0.001]. ANOVA, comparing mean performance before and after surgery, revealed no effect of cannulae implantation on performance [*F*_(1,7)_ 1.8, *p* < 0.2; **Figure 9**].

##### Test session

Temporary inactivation of the retrosplenial cortex with muscimol severely disrupted performance on the standard T-maze task (**Figure 9**). ANOVA, comparing performance under muscimol or the PBS control infusion, revealed an effect of infusion [*F*_(1,7)_ = 90.2, *p* < 0.0.001]. While performance under PBS was reliably above chance [*t*_(7)_, = 16.5, *p* < 0.001], performance was at chance following temporary inactivation of the retrosplenial cortex [*t*_(7)_ = 1.2, *p* = 0.3].

## Discussion

Consistent with previous findings (e.g., Pothuizen et al., 2008), the extensive retrosplenial cortex lesions in the current study produced only mild impairments on standard T-maze alternation (‘standard,’ Experiment 1, Stage 1). Moreover, the impairment was transient – with more extensive training the lesioned animals performed at levels equivalent to the Sham group on the standard alternation task [(Experiment 1, Stage 3; Experiment 2 Stage 2 and Stage 3 pre-training; see also (Neave et al., 1994; Aggleton et al., 1995)]. The experiments reported here examined three possible explanations for this apparent sparing of T-maze performance following retrosplenial lesions and considered how this sparing casts light on retrosplenial cortex function.

Animals have available multiple classes of spatial information with which to solve alternation tasks (e.g., Dember and Fowler, 1958; Dudchenko, 2001; Futter and Aggleton, 2006). Consequently, one plausible explanation is that retrosplenial cortex lesions only disrupt a subset of strategies, often allowing the rats to perform at near-normal levels. Consequently, we sought to assess the impact of retrosplenial cortex lesions on T-maze alternation by altering the availability of cue types. The most striking and consistent retrosplenial lesion impairment occurred when the maze was rotated between the sample and choice phases (found in both lesion cohorts: Experiment 1, Stage 2; Experiment 2, Stages 1–2; see **Figures 4** and **6**). The same intra-trial delay (10 s) was used across all trial types and the maze rotation occurred once the rat had been removed from the sample arm. Thus, the sensitivity to maze rotation was not due to any greater disruption of the animals or to longer retention periods. These selective lesion effects on intra-trial maze rotation are also consistent with studies using the eight-arm radial arm maze. Here, rats with retrosplenial lesions often appear unaffected during normal training, but when the maze is rotated after four correct arm choices (placing intra- and extra- maze cues in conflict), performance is excessively disrupted (Vann et al., 2003; Vann and Aggleton, 2004; Pothuizen et al., 2008). Together, these deficits indicate that retrosplenial cortex lesions either leave rats unusually reliant on intra-maze cues or less able to shift from this cue class when it proves misleading.

A corollary of the first explanation (excessive reliance on intra-maze cues) is that rats with retrosplenial lesions are less likely to use distal allocentric or directional cues. The retrosplenial cortex, and in particular the dysgranular subregion, is densely connected with cortical areas (e.g., areas 17 and 18b) that provide visual information (van Groen and Wyss, 1992), consistent with this suggestion. Support for this prediction came from when the sample phase was in the light but the choice phase switched to the dark (Experiment 1, Stage 3). Now, rats with retrosplenial cortex lesions outperformed the controls over the first block of trials (**Figure 5**), presumably reflecting their excessive reliance on non-visual intra-maze cues, which could still guide spatial alternation. Other evidence concerning the relative use of distal visual cues comes from Morris water-maze studies, which show that retrosplenial lesions can impair place learning, whether acquired by actively swimming to an escape platform (e.g., Sutherland et al., 1988; Warburton and Aggleton, 1998; Vann and Aggleton, 2002) or by merely being placed on the platform (Nelson et al., 2015). Further retrosplenial lesion evidence indicates a difficulty in using distal spatial cues comes from tasks involving the discrimination of room locations (Hindley et al., 2014b).

Despite these consistent findings, the present study also shows that difficulties with using distal visual cues does not provide a full account of the effects of retrosplenial cortex lesions. One example concerns those alternation trials with a sample in the light and a choice trial in the dark (Experiment 1, Stage 3). Despite an initial advantage, the rats with retrosplenial cortex lesions failed to improve over successive trials, even though a reliance on intra-maze cues should provide an effective strategy. Another inconsistency comes from Experiment 2 stage 3, which used two adjacent cross-mazes, one for the sample and one for the choice test. Now, there was little or no evidence of a retrosplenial lesion impairment when animals were tested in two adjacent mazes in the light, despite the apparent need to rely on distal visual cues (**Figure 8**).

A potential limitation with the two-maze task (Experiment 2, stage 3) may arise from the extent to which animals appreciate the use of two rather than one maze, each in different locations. It is also possible that Sham rats are more aware of this change, e.g., because of greater sensitivity to different locations within the test room (Hindley et al., 2014b). Evidence that confusion associated with an awareness of the two mazes, which could drive down performance, came from the improvement when tested in two mazes in the dark (**Figure 8**). Even so, a lesion impairment still emerged when testing in the two mazes occurred in the dark. Clearly, the availability of distal visual cues is not a sufficient condition to produce a retrosplenial cortex lesion deficit. Instead, the pattern of results suggests that in the absence of intra-maze cues in the light, retrosplenial lesion animals are able to use other sources of spatial information (e.g., allocentric visual cues) to support spatial alternation, but when forced to rely on directional information in the dark, a retrosplenial lesion impairment emerges (Pothuizen et al., 2008). These data further suggest that although retrosplenial damage disrupts the use of both directional and allocentric information, these impairments are not absolute. Although impaired relative to sham animals, retrosplenial performance in the double maze in the dark was still consistently above chance levels (Experiment 2, stage 3; **Figure 8**). Similarly, when the trial type remained constant across sessions and the value of both allocentric and intra-maze cues was nullified in the dark with maze rotation (Experiment 2, Stage 2; **Figure 7**), performance in the retrosplenial group remained above chance levels. As animals are unable to use egocentric response strategies to alternate (Futter and Aggleton, 2006; Pothuizen et al., 2008), this leaves either directional or idiothetic cues as the only available cues under these testing conditions. Although the retrosplenial cortex has been implicated in some forms of idiothetic learning in the dark (e.g., Cooper et al., 2001; Elduayen and Save, 2014), the finding that retrosplenial lesion animals are still able to perform above chance levels in the absence of both extra- and intra-maze cues suggests that, in highly constrained environments such as the T-maze, retrosplenial damage does not preclude entirely the use of idiothetic or directional information to guide alternation performance (see also Zheng et al., 2003). Further evidence supporting this conclusion comes from the finding that retrosplenial lesions only moderately impair landmark control over head-direction cells in the anterodorsal thalamic nucleus (Clark et al., 2010). The reduced stability of these cells’ preferred directional firing may affect the ability to alternate when reliant on directional cues (Dudchenko, 2001), with alternation performance remaining near-normal for standard training.

The inference is that the disruptive effects of maze rotation on retrosplenial performance are not solely due to a failure to use distal visual cues effectively. This conclusion leads to the second explanation, that the retrosplenial cortex helps the animal switch between competing strategies, e.g., competing cue types. With this explanation in mind, it is helpful to appreciate that maze rotation does not simply negate the value of intra-maze cues, it places them in conflict with other available cue types. To perform the T-maze task effectively under these circumstances, animals must presumably ignore the conflicting information and rely on those cue types most reliably associated with correct performance. The finding that T-maze performance in the Sham animals was barely affected by maze rotation in the light suggests that normal animals are rapidly able to disregard conflicting intra-maze cues and rely more on extra-maze cues to support effective spatial alternation. Consistent with this suggestion, Sham group performance dropped to levels observed in the retrosplenial lesion group when the maze was rotated in the dark and the use of allocentric cues was explicitly excluded, i.e., both intra- and extra- maze cues were removed (**Figure 6**).

As already alluded, one hypothesis is that the retrosplenial cortex serves as a comparator that translates spatial representations from one frame of reference to another (Burgess, 2002; Byrne et al., 2007). More broadly, the retrosplenial cortex has been implicated in forming associations between multiple cues and outcomes (e.g., Robinson et al., 2011), encoding behaviorally significant outcomes (e.g., Smith et al., 2012) and processing the relationship between cues (for a review see Miller et al., 2014). Such a function would only be engaged for T-maze alternation when animals are required to switch between different spatial strategies (Vann et al., 2009). Support for this view comes from findings that the retrosplenial lesion impairments on spatial memory tasks may only emerge when animals are required to switch between competing strategies (Wesierska et al., 2009) or there is a change from light to dark conditions during testing (Cooper and Mizumori, 1999). Indeed, recent evidence points to a wider role for the retrosplenial cortex in translating between different representations of the same event, whether in the spatial (e.g., Nelson et al., 2015) or non-spatial (Hindley et al., 2014a; Nelson et al., 2014) domain. In the present study, switching away from intra-maze cues should, therefore, be particularly difficult.

The apparent initial failure of the Sham animals to switch from using allocentric frames of reference to alternative sources of spatial information (e.g., directional, idiothetic or intra-maze) might suggest that intact animals are not always able to switch spontaneously between strategies, at least on a trial by trial basis (see Experiment 1, Stage 3; Experiment 2, Stage 3). In both experiments, the animals were also subject to changes in cue type (within session in Experiment 1; across session in Experiment 2, Stage 3). Nevertheless, as training progressed, performance in the Sham animals recovered and was consistently above chance. In contrast, there was no equivalent improvement in the RSC animals. Thus, Sham animals were clearly able to adopt other available strategies (e.g., directional, intra-maze, idiothetic) to support spatial alternation. The lack of improvement in the retrosplenial cortex lesion animals is indicative of a less flexible use of the various, available sources of spatial information.

A third explanation for seemingly intact T-maze alternation levels after retrosplenial cortex lesions presumes that compensatory mechanism in other structures partially mask retrosplenial involvement. We, therefore, temporarily inactivated the retrosplenial cortex with muscimol. In contrast to the effects of permanent lesions, temporary inactivation of the retrosplenial cortex resulted in a profound T-maze alternation deficit, comparable to that found after hippocampal or anterior thalamic nuclei damage (Aggleton et al., 1986, 1996; Béracochéa and Jaffard, 1994; Bannerman et al., 1999; Dudchenko et al., 2000). This apparent dissociation between the effects of temporary and permanent lesions has been reported previously, albeit with tetracaine (Cooper and Mizumori, 2001). By using muscimol, a potent GABA_A_ agonist that spares axons, the current study rules out effects on fibers of passage. The present deficit shows how the importance of the retrosplenial cortex for spatial alternation can be masked by compensatory mechanisms, reflecting the parallel involvement of closely related structures. One prediction that follows is that this deficit should abate with repeated testing under muscimol as this testing regime would allow functional compensation by other structures to emerge. This compensation presumably involves the use of other spatial metrics such as intra-maze cues to solve the alternation task. As such, the results of Experiment 3 are also consistent with the notion that the retrosplenial cortex aids the flexible use of multiple spatial strategies. If, as would be predicted on the basis of the lesion data, temporarily silencing the retrosplenial cortex disrupts the use of allocentric and directional information, the animal is forced to rely on other classes of spatial information. Thus, one explanation for the profound effects of retrosplenial inactivation on alternation performance is that it compounds the deficit in allocentric and directional information by preventing the animal from switching to alternative strategies.

The current study had three main goals. The first was to determine whether the retrosplenial cortex is important for just one cue type and the second goal was to test the specific hypothesis that the retrosplenial cortex helps the animal switch between competing strategies or competing cue types. One clear finding was that retrosplenial lesions do not preclude all spatial strategies. Consistent deficits only emerged when animals were forced to rely on directional information in the dark or when allocentric and intra-maze cues were placed in conflict. As has been found previously (Vann and Aggleton, 2002; Pothuizen et al., 2008), these impairments in the use of directional and allocentric information are not absolute. Evidence also emerged that an inability to switch flexibly between different classes of spatial information may contribute to these effects. On the basis of the lesion data alone, it is not possible to dissociate these positions, which are not mutually exclusive. Indeed, given the failure of the control animals to adapt flexibly to intra-trial changes in the availability of spatial cues (e.g., Experiment 1, Stage 3), it may not be possible to dissociate these positions empirically. Similarly, as retrosplenial lesions seemingly disrupt the use of both directional and allocentric information it is difficult to conclude with any certainty when the observed deficits arise from the impoverished use of these classes of information or a failure to adapt alternative spatial strategies. In this respect, the demonstration that temporary inactivation of the retrosplenial has a profound effect on standard T-maze alternation is particularly revealing.

## Conflict of Interest Statement

The authors declare that the research was conducted in the absence of any commercial or financial relationships that could be construed as a potential conflict of interest.
